# Protein-Related Dietary Parameters and Frailty Status in Older Community-Dwellers across Different Frailty Instruments

**DOI:** 10.3390/nu12020508

**Published:** 2020-02-17

**Authors:** Hélio J. Coelho-Júnior, Riccardo Calvani, Anna Picca, Ivan O. Gonçalves, Francesco Landi, Roberto Bernabei, Matteo Cesari, Marco C. Uchida, Emanuele Marzetti

**Affiliations:** 1 Università Cattolica del Sacro Cuore, 00168 Rome, Italy; riccardo.calvani@guest.policlinicogemelli.it (R.C.); francesco.landi@unicatt.it (F.L.);; 2 Applied Kinesiology Laboratory-LCA, School of Physical Education, University of Campinas, 083-851 Campinas-SP, Brazil; uchida@unicamp.br; 3 Mãe Mariana Nursing Home, Rehabilitation unit, 08562-460 Poá-SP, Brazil; 4 Fondazione Policlinico Universitario “Agostino Gemelli” IRCCS, 00168 Rome, Italy; 5 Center of Health Sciences, University of Mogi das Cruzes, 08780-911 Mogi das Cruzes, Brazil; ivanogedfisica@gmail.com; 6 Department of Clinical Sciences and Community Health, Università di Milano, 20133 Milan, Italy; 7 Geriatric Unit, Fondazione IRCCS Ca’ Granda Ospedale Maggiore Policlinico, 20122 Milan, Italy

**Keywords:** aging, diet, physical function, disability, sarcopenia, nutrition, amino acids, metabolism, dietary patterns, protein per meal

## Abstract

The present study investigated the associations between frailty status and (a) daily protein intake, (b) daily body weight-adjusted protein intake, (c) branched-chain amino acid (BCAA) consumption, (d) evenness of protein distribution across main meals, (e) number of daily meals providing at least 30 g of protein, and (f) number of daily meals providing at least 0.4 g protein/kg of body weight in community-dwelling older adults. The relationship between frailty status and protein-related dietary parameters was explored across different frailty assessment tools. Two hundred older adults were enrolled in the study. Participant frailty status was determined according to a modified Fried’s frailty phenotype (mFP), the FRAIL scale, and the Study of Osteoporotic Fracture (SOF) index. Diet was assessed by 24-h dietary recall, while diet composition was estimated using a nutritional software. A frailty instrument-dependent relationship was observed between frailty status and protein-related dietary parameters. Protein consumption was associated with frailty status only in participants identified as frail according to the mFP. In addition, protein and BCAA intake was found to be greater in robust and pre-frail participants relative to their frail counterparts. Our findings suggest that the association between frailty and protein-related dietary parameters is tool dependent. Specifically, protein and BCAA consumption appears to be lower only in older adults identified as frail by the mFP.

## 1. Introduction

Frailty is a highly prevalent condition in older adults [1] and is defined as a state of increased vulnerability to negative health-related outcomes, which occurs as a result of multisystem derangements and poor social support [2,3,4]. Frailty increases the risk for many adverse events, including falls and fractures, disability, hospitalization, nursing home placement, and death [5,6]. Although substantial agreement exists on the theoretical construct of frailty, its clinical implementation is hampered by the lack of an unequivocal operational definition. This impasse is reflected by the existence of numerous instruments for frailty assessment, and each of these identifies only partially overlapping phenotypes [7,8].

The phenotypic model of frailty was developed and operationalized by Fried et al. [9] using data from the Cardiovascular Health Study (CHS). The frailty phenotype (FP) is based on five elements: (1) unintentional weight loss; (2) dynapenia; (3) fatigue; (4) poor mobility; and (5) inactive lifestyle. The assessment is quantitative such that people with three or more factors are classified as frail, while those with one or two factors are considered pre-frail. Ensrud et al. [10] have argued that the clinical relevance of the FP is limited by the fact that cutoffs for handgrip strength, walking speed, and sedentary behavior were established based on population data. As an attempt to overcome this possible limitation, the Study of Osteoporotic Fractures (SOF) index was designed and validated based on the self-report of weight loss, inability to rise from a chair five times without using the arms, and reduced energy levels [10]. More recently, Morley et al. [11] proposed the FRAIL scale, which involves five self-reported items (fatigue, resistance, ambulation, illness, loss of weight), to allow identification of frailty without a face-to-face examination. Although these three screening instruments have shown comparable performance in predicting negative health outcomes [12,13,14], the relationship between health determinants and frailty status across these tools is presently unclear.

“Healthy” diet is a key factor for preserving independence during aging [15,16,17]. In particular, a high protein intake has been associated with better physical performance [18,19] and lower prevalence of frailty [20,21,22,23,24]. These observations have prompted expert groups and scientific societies to recommend that older adults increase their protein intake above the recommended dietary allowance (RDA) [25,26,27,28]. The impact of dietary protein on frailty is believed to be mainly related to the action of branched chain amino acids (BCAAs) on skeletal muscle [29]. Indeed, adequate amounts of BCAAs, in particular leucine, are essential to stimulate muscle protein synthesis (MPS) [29,30].

Previous research in the field has mostly focused on the effects of protein intake as a whole on frailty-related parameters. However, recent evidence suggests that the distribution of protein across meals may be more important than the total amount of protein ingested during the day [31,32,33]. Remarkably, older adults who reported a spread-feeding pattern in which dietary protein was evenly distributed across the main meals had faster gait speed than those who followed a pulse-feeding pattern (i.e., high protein intake in a single meal) [33]. Moreover, Loenekke et al. [31] found that older adults consuming meals containing ≥30 g of protein had greater lower-limb muscle strength and leg lean mass. This finding led to the assumption that a minimum of 30 g of protein per meal may be necessary to maintain physical performance and muscle mass in old age. This view is supported by the observation that MPS is maximally stimulated by the ingestion of 0.4 g of high-quality dietary protein/kg of body weight in older men [34,35].

The presence of heterogeneous recommendations about dietary protein regimens implies that additional information is necessary to develop nutritional solutions to promote robustness in old age. The present study investigated the relationship among frailty status, which was assessed by three different screening tools, i.e., a modified FP (mFP), SOF and FRAIL, and (a) daily protein consumption, (b) daily body weight-adjusted protein consumption, (c) daily BCAA intake, (d) evenness of protein distribution across the three main meals (i.e., breakfast, lunch, dinner), (e) number of daily meals providing at least 30 g of protein, and (f) number of daily meals providing at least 0.4 g of protein/kg of body weight in a convenience sample of community-dwelling older persons.

## 2. Materials and Methods

The study protocol was approved by the Research Ethics Committee of the University of Campinas (Campinas, Brazil) under the protocol number 621–614. All study procedures were conducted in compliance with the Declaration of Helsinki and Resolution 196/96 of the National Health Council. Participants were thoroughly informed about the study procedures and objectives before they provided written consent.

### 2.1. Study Participants

Participants were recruited from a community senior center located in Poá, Brazil. Poá is a city located in the southern area of São Paulo, with a population of approximately 100,000 people of whom ~3460 are 60+ years old [36]. The community senior center offers daily sessions for flexibility, aquatic and multicomponent physical exercises, dance classes, adapted sports, nursing and medical care, and cognitive stimulation therapy. Candidate participants were considered eligible if they were 60 years or older, lived in the community, and possessed sufficient physical and cognitive abilities to perform all of the measurements required by the protocol. Candidates were excluded if they were on hormone replacement therapy and/or psychotropic drugs.

### 2.2. Disease Conditions

Information pertaining to disease conditions was collected by two researchers (H.J.C.-J. and I.O.G.) through self-report and careful review of medical charts from the community senior center. Medical charts, which are updated every six months by a local physician, were reviewed to determine the prevalence of disease conditions that may impact physical performance (e.g., osteoarthritis) or nutrition (e.g., gastrointestinal diseases).

### 2.3. Assessment of Frailty Status

#### 2.3.1. Modified Frailty Phenotype

The FP, operationalized by Fried et al. [9], incorporates measures of multiple physical domains, including weight loss, exhaustion, weakness, slowness, and sedentary behavior [14,37]. People are classified as robust, pre-frail, and frail depending on to the presence of none, 1–2, and ≥3 defining criteria, respectively. In the present study, the following indicators were used to compute a mFP: (1) unintentional weight loss ≥5 kg in the previous year; (2) self-reported fatigue [i.e., a response of “a moderate amount of the time (3–4 days)” or “most of the time” to the Center for Epidemiologic Studies Depression Scale (CES-D) item: “I felt that everything I did was an effort” during the past week]; (3) weakness, operationalized as a handgrip strength normalized to body mass index (BMI) < 0.8; (4) slowness, defined by a timed “up-and-go” (TUG) performance ≥6.7 s [38]; and (5) low physical activity levels according to gender-specific cutoffs of the short form of the International Physical Activity Questionnaire (IPAQ) [14,39].

#### 2.3.2. FRAIL Scale

The FRAIL scale consists of five simple questions requiring a yes/no answer, with one point assigned to any affirmative response [40]. Possible scores range from 0 to 5 points, and people are classified as robust (0 points), pre-frail (12 points), and frail (≥3 points) according to the following criteria: (1) self-reported fatigue; (2) poor resistance, based on the inability to climb a flight of stairs; (3) limited ambulation, based on the inability to walk one block; (4) illnesses, presence of ≥5 illnesses; and (5) unintentional weight loss of ≥5% in the past six months.

#### 2.3.3. Study of Osteoporotic Fracture Index

The SOF index is based on three criteria: (1) unintentional weight loss of ≥4.5 kg in the prior year; (2) self-reported exhaustion; and (3) inability to rise from a chair five times without using arms. SOF scores range from 0 to 3, and people are identified as robust, pre-frail, and frail according to the presence of 0, 1, and 2–3 criteria, respectively [10].

### 2.4. Dietary Assessment

Food intake was assessed by 24-h dietary recall [41]. The method uses an open-ended questionnaire to provide a quantitative and subjective estimation of actual food consumption. In the present study, two trained researchers (H.J.C.-J. and I.O.G.) asked the participants to recall in detail all foods they consumed on a meal-by-meal basis, including snacks, during the previous 24 h. Interviews occurred on Tuesdays, Wednesdays, Thursdays, and Fridays to avoid possible biases associated with the weekend. Participants were requested to provide details about cooking methods (e.g., fried, grilled, roasted), serving and portion sizes, product brands, sauces, spices, and condiments consumed, and the use of dietary supplements. Amounts of beverages consumed were also recorded, and participants were asked to specify if and how beverages were sweetened. Two-dimensional aids (e.g., photos), household utensils (e.g., standard measuring cups, spoons), and food models were used to facilitate assessment of portion sizes. Diet composition was estimated using the NutWin software, version 1.5 (Federal University of São Paulo, Brazil) [18].

### 2.5. Statistical Analysis

Continuous and categorical variables were compared among the three groups (i.e., robust, pre-frail, and frail) via one-way analysis of variance (ANOVA) and χ^²^ statistics, respectively. Bonferroni post hoc analyses were performed to determine whether there were significant differences between groups. Agreement among frailty screening tools was evaluated by κ statistics. Chi-squared and Z-score were further used to explore the association between diet characteristics and frailty status within frailty instruments. Median values were chosen as the cutoff values for isoleucine (4.4 g), leucine (7.1 g), and valine (4.7 g) intakes. Cutoffs for body weight-adjusted daily protein consumption [19,24], body weight-adjusted protein consumption per meal [34], and protein consumption per meal [31] were chosen based on previous reports. Protein intake distribution across the main meals (i.e., breakfast, lunch, and dinner) was calculated for each participant as a coefficient of variance (CV) [33], as follows:

CV = standard deviation of g of protein intake per main meal/average total amount of protein (g) of the main meals(1)

Participants were stratified in tertiles according to CV values (<0.38, 0.45, and >1.0). Low CV values correspond to a more even distribution of protein intake across meals and are therefore indicative of a more spread pattern. High CV values, instead, reflect greater variability in per-meal protein intake, indicating a pulse-feeding distribution of protein ingestion [33]. For all tests, alpha was set at 5% (*p* < 0.05) and Z-score was set at 1.96. All analyses were conducted using the IBM SPSS Statistics, version 20.0, software (IBM Corp., Armonk, NY, USA).

## 3. Results

### 3.1. Characteristics of Study Participants

**Figure 1:**
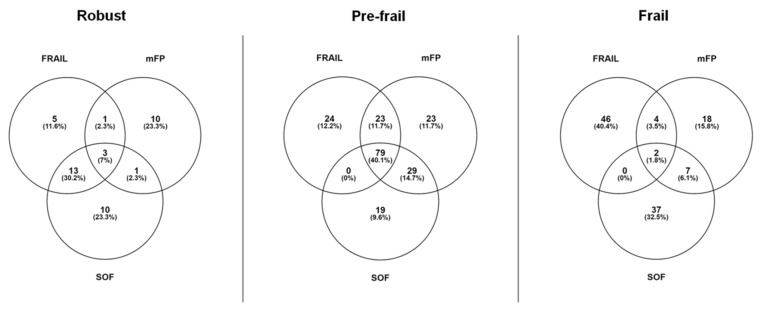
Venn diagram showing the extent of overlap of frailty categories among the three frailty screening tools used. Abbreviations: mFP, modified frailty phenotype; SOF, Study of Osteoporotic Fractures.

Two hundred fifty-four people agreed to be evaluated for inclusion. Of these, 46 were younger than 60 years, six had missing data for frailty status, and two had missing data for diet, leaving a total of 200 participants. The main characteristics of study participants according to frailty status and assessment instruments are shown in Table 1. The prevalence of frailty was 15.5% according to the mFP, 23.0% using the SOF index, and 26.0% according to the FRAIL scale. A moderate agreement was observed between mFP and FRAIL (κ = 0.696; *p* < 0.001) and between mFP and SOF (κ = 0.526; *p* < 0.039), while a high agreement (κ = 0.951; *p* < 0.001) was determined between FRAIL and SOF (Figure 1). 

Sociodemographic and clinical characteristics as well as protein intake per meal were comparable across frailty statuses regardless of the assessment tool considered. Similarly, physical activity levels, total daily protein and BCAA intake did not vary across the frailty categories identified by the FRAIL scale and the SOF index. On the other hand, significant differences in anthropometric characteristics, physical activity levels, daily protein consumption, and BCAA intake across mFP categories were observed. Specifically, pre-frail participants were heavier than their robust counterparts (*p* = 0.034). Frail individuals reported lower daily consumption of protein (*p* = 0.005), isoleucine (*p* = 0.004), leucine (*p* = 0.004), and valine (*p* = 0.005) compared with both pre-frail and robust participants, as well as lower physical activity levels relative to pre-frail (*p* = 0.001), but not frail participants (*p* = 0.383). In addition, body weight-adjusted daily protein intake in frail persons was lower than in their robust peers. No significant differences in dietary protein-related parameters were observed among the frailty categories identified by the three frailty assessment tools.

### 3.2. Participant Distribution According to Dietary Parameters and Frailty Status

The relationship between dietary protein categories and frailty status across the three assessment tools used is shown in Table 2. No significant differences in participant distribution across protein consumption categories, including patterns of protein consumption across main meals, were observed regardless of the frailty assessment tool considered. Instead, a significant association was found between daily ingestion of isoleucine, leucine, and valine and the frailty categories defined by the mFP (all *p* values < 0.001). Indeed, a greater proportion of pre-frail participants reported an intake greater than the median values for all three BCAAs. An opposite pattern was detected in frail individuals.

## 4. Discussion

The present study investigated the relationship between frailty status and several dietary protein-related parameters across three popular frailty screening tools in a sample of community-dwelling older adults. Our findings indicate that the relationship between characteristics of dietary protein intake and frailty is tool-dependent. Indeed, significant associations between frailty status and protein consumption were only observed when frailty was assessed though a mFP. Specifically, frail participants reported lower daily consumption of protein and BCAAs compared with both pre-frail and robust individuals.

Previous studies have explored the relationship between frailty status assessed by different instruments and cognitive function, falls, disability, fractures, hospitalization, and all-cause mortality [12,13,14]. Frailty assessment tools showed similar performance in predicting negative outcomes, even when frailty was operationalized with different constructs (e.g., physical frailty and deficit accumulation paradigm) [12,13,14]. However, in pre-frail older adults, the incidence of disability and hospitalization was predicted differently by the various instruments, such that the FRAIL scale and the SOF index were associated with disability, whilst the FP and the SOF index were predictive of hospitalizations [14]. This observation suggests that the pathogenic processes associated with pre-frailty progression might be differentially captured by individual frailty assessment tools.

In the present study, protein intake-related parameters were significantly associated with the frailty status when using a mFP, but not FRAIL or SOF. One possible explanation for this finding may reside in the methods used to assess physical function by the various instruments. Indeed, protein intake has a key role in stimulating MPS and, consequently, in the preservation of muscle mass and physical performance [30,42]. While physical function is objectively measured in the mFP, the evaluation of the functional domain in FRAIL and SOF relies on self-report, which does not necessarily reflect the actual physical performance status [43,44].

Studies have demonstrated a weak-to-moderate correlation between self-reported and objectively measured physical function in older adults [45,46,47,48,49] and in those with chronic degenerative diseases [50,51]. Indeed, a systematic-review of studies that investigated only older adults indicated that the coefficient of correlation between subjective and objective physical performance measures ranged from −0.72 to 0.71 [52]. This lack of concordance may be explained by the fact that subjective measures reflect people’s perception of their own environment [52] and are more influenced by the presence of diseases, pain, joint stiffness, sociocultural background, use of medications, depressive symptoms, and cognitive function [45,46,47,48,49,50,51,52]. Hence, although both valid, subjective and objective measures capture different aspects of physical function.

Along similar lines, an inverse relationship between BCAA consumption and frailty status was observed, but only according to the mFP. Contrary to frail individuals, most robust and pre-frail participants reported intakes of isoleucine, leucine, and valine greater than the respective median values. These findings are in keeping with previous investigations reporting a negative association between frailty and intake of essential amino acids [21,53]. For instance, Beasley et al. [21] found that a higher intake of essential amino acids was significantly associated with lower risk of developing frailty over three years in a subset of older women enrolled in the Women’s Health Initiative Observational Study (WHI-OS). Similarly, Kobayashi et al. [53] found that a greater consumption of selected amino acids, including BCAAs, was associated with lower prevalence of frailty in a large sample of Japanese older women.

Our findings add to the current knowledge on the relationship between amino acid intake and frailty and suggest that the association may be influenced by BCAA ingestion. According to the anabolic resistance hypothesis [54], the stimulation of MPS is blunted in advanced age, which contributes to loss of lean body mass, dynapenia, and impaired physical function [19,54]. A greater intake of amino acids, especially leucine, is believed to be necessary to overcome the anabolic resistance and appropriately stimulate MPS [30]. This view is supported by observational studies which found that reduced systemic concentrations of BCAAs were significantly associated with sarcopenia markers in older adults [55,56].

The results of the present study do not support the hypothesis that protein consumption across main meals is associated with frailty status in older adults. Previous studies found inconsistent results regarding the association between protein eating patterns per meal and frailty-associated parameters. Loenneke et al. [31] reported that community-dwelling older adults who consumed two or more meals containing ≥ 30 g of protein had greater knee strength and leg lean mass than those who did not consume any meal with at least 30 g of protein. Similarly, Farsijani et al. [57] and Ten Haaf et al. [33] found better physical performance in older adults who had a protein intake spread across the main meals. Furthermore, women with a low skeletal muscle index (SMI) were found to ingest lower amounts of animal protein at breakfast in comparison with those with a high SMI [32]. However, no significant associations among protein eating patterns, muscle mass, and physical function were determined in successful agers [58]. Only one study investigated whether frailty was associated with protein intake across meals. In this report, Bollwein et al. [59] found that robust older adults showed a more even protein eating pattern than pre-frail and frail people.

Discrepancies among studies may be attributed to sample characteristics, including age, sex distribution, and dietary patterns. Regardless of frailty status, participants of the present study showed higher average body mass-adjusted protein intake in comparison with values reported by Bollwein et al. [59]. Furthermore, we found that most frail participants consumed at least one meal containing ≥ 30 g of protein. Notably, the association of protein intake with muscle mass and physical performance plateaus at approximately 30 g of protein per meal [31].

The present study has some limitations that should be acknowledged. The study population was relatively small, young, physically active, and with a high prevalence of participants whose protein and BCAA intakes were higher than RDA values. Unexpectedly, a high body weight-adjusted protein intake (1.3 g/kg) was found in frail participants regardless of the frailty screening tool considered. This may be due to the fact that participants were recruited in a community center for older adults, where they receive advice on healthy lifestyle habits, including nutrition. In addition, the study sample included only community-dwellers. Hence, our findings should be replicated in other settings as well as in older adults with less access to health information. Frailty usually develops as a result of a long-term process. Therefore, the assessment of diet over short periods of time (e.g., 24 h, seven days) may not capture the possible contribution of eating behaviors to the genesis and progression of frailty. The FP was adapted given that the usual walking speed test was replaced by TUG [60]. In addition, only screening tools were used for frailty assessment. Thus, our findings should be confirmed using multidimensional frailty instruments (e.g., frailty index, clinical frailty scale) [61]. Finally, the cross-sectional design of the study does not allow any inference to be drawn on the time course of changes in the variables considered and on cause-effect relationships.

## 5. Conclusions

Collectively, the findings from the present study indicate that the relationship between diet characteristics and frailty status may be dependent upon the frailty assessment tool used. Indeed, a significant association between frailty and protein-related dietary parameters was only observed when the frailty status was assessed through a mFP. This observation suggests that frailty instruments including performance-based measures of physical function should be preferentially used when designing nutritional interventions for older adults and to evaluate the impact of dietary habits on frailty status.

## Figures and Tables

**Table 1:** Characteristics of study participants according to frailty status across frailty assessment instruments.

FRAIL				mFP			SOF		
Variables	Robust (*n* = 22)	Pre-Frail (*n* = 126)	Frail (*n* = 52)	Robust (*n* = 15)	Pre-Frail (*n* = 154)	Frail (*n* = 31)	Robust (*n* = 27)	Pre-Frail (*n* = 127)	Frail (*n* = 46)
*General Characteristics*									
Age, years	66.0 ± 4.5	68.8 ± 7.3	67.6 ± 6.4	65.1 ± 7.5	67.8 ± 6.1	71.2 ± 9.1	68.6 ± 8.0	68.2 ± 6.9	68.0 ± 6.1
Body weight, kg	66.5 ± 11.7	69.6 ± 12.7	68.2 ± 10.4	63.5 ± 9.2	69.9 ± 12.1 ^a^	66.4 ± 12.4	68.8 ± 12.2	69.7 ± 12.0	66.7 ± 12.0
BMI, kg/m^2^	28.1 ± 4.4	28.9 ± 5.3	28.5 ± 4.3	27.7 ± 3.6	28.9 ± 5.0	28.4 ± 5.5	29.4 ± 5.2	29.0 ± 4.8	27.5 ± 5.1
Physical activity levels, min/week	490.7 ± 112.3	487.5 ± 92.4	473.4 ± 100.0	476.4 ± 81.9	497.1 ± 88	430.0 ± 123.2 ^b^	490.7 ± 112.3	487.5 ± 92.4	473.4 ± 100.6
Sex, female	18 (81.8)	103 (81.7)	45 (86.5)	13 (92.9)	125 (81.2)	27 (87.1)	22 (81.5)	106 (83.5)	38 (82.6)
Current smokers	0 (0.0)	3 (2.4)	3 (5.8)	1 (7.1)	4 (2.6)	1 (3.2)	0 (0.0)	3 (2.4)	3 (6.5)
Multimorbidity	0 (0.0)	7 (5.6)	7 (13.5)	2 (14.3)	6 (3.9)	6 (19.4)	4 (14.8)	9 (7.1)	1 (2.2)
*Ethnicity*									
Asian	0 (0.0)	8 (6.3)	3 (5.8)	0 (0.0)	9 (5.8)	2 (6.5)	0 (0.0)	7 (5.5)	4 (8.7)
Black	3 (13.6)	22 (17.5)	12 (23.1)	2 (14.3)	28 (18.2)	7 (22.6)	6 (22.2)	22 (17.3)	9 (19.6)
Caucasian	19 (86.4)	96 (76.2)	37 (71.2)	12 (85.7)	117 (76.0)	22 (71.0)	21 (77.8)	98 (77.2)	33 (71.7)
*Diet*									
Protein, g	112.6 ± 47.3	105.4 ± 40.8	104.9 ± 38.7	122.2 ± 37.3	108.2 ± 41.4	89.7 ± 36.4 ^a,b^	106.4 ± 43.8	107.4 ± 41.4	102.4 ± 38.3
Protein, g/kg	1.7 ± 0.8	1.5 ± 0.6	1.5 ± 0.5	1.9 ± 0.6	1.5 ± 0.6	1.3 ± 0.4 ^a^	1.6 ± 0.7	1.5 ± 0.6	1.5 ± 0.5
Valine, g	5.5 ± 2.3	5.2 ± 2.1	5.3 ± 2.1	6.1 ± 1.9	5.4 ± 2.1	4.4 ± 1.9ab	5.2 ± 2.1	5.3 ± 2.1	5.0 ± 2.0
Isoleucine, g	4.6 ± 2.9	4.7 ± 2.3	4.7 ± 2.0	5.3 ± 2.2	4.8 ± 2.3	3.9 ± 1.9ab	4.4 ± 2.7	4.8 ± 2.3	4.6 ± 1.9
Leucine, g	8.4 ± 3.5	7.9 ± 3.2	7.9 ± 3.0	9.3 ± 2.9	8.2 ± 3.2	6.7 ± 2.9ab	7.9 ± 3.2	8.1 ± 3.2	7.7 ± 3.0
Protein breakfast, g	11.4 ± 5.2	13.3 ± 7.1	12.9 ± 7.4	12.8 ± 5.2	12.9 ± 7.7	12.6 ± 7.1	11.7 ± 4.7	13.1 ± 7.1	12.8 ± 9.4
Protein lunch, g	59.5 ± 33.6	54.8 ± 26.4	58.5 ± 28.4	70.2 ± 28.4	56.2 ± 28.5	50.8 ± 22.0	56.4 ± 30.8	56.3 ± 28.1	56.3 ± 27.7
Protein dinner, g	31.0 ± 29.7	23.9 ± 27.2	21.7 ± 25.7	28.3 ± 30.9	25.8 ± 27.7	14.9 ± 21.0	25.4 ± 29.3	24.8 ± 27.6	21.6 ± 24.8
Protein breakfast, g/kg	0.17 ± 0.09	0.19 ± 0.10	0.17 ± 0.12	0.20 ± 0.09	0.18 ± 0.11	0.18 ± 0.10	0.17 ± 0.81	0.19 ± 0.11	0.18 ± 0.11
Protein lunch, g/kg	0.90 ± 0.55	0.81 ± 0.41	0.86 ± 0.41	1.1 ± 0.46	0.82 ± 0.44	0.76 ± 0.30	0.82 ± 0.50	0.83 ± 0.43	0.85 ± 0.3.
Protein dinner, g/kg	0.49 ± 0.49	0.35 ± 0.40	0.30 ± 0.37	0.46 ± 0.51	0.38 ± 0.41	0.20 ± 0.29	0.40 ± 0.48	0.36 ± 0.41	0.30 ± 0.37

Data are presented as means ± standard deviations, except for sex, current smokers, multimorbidity (i.e., ≥5 diseases), and ethnicity, which are shown as absolute numbers and percentages. Abbreviations: BMI, body mass index; mFP, modified frailty phenotype; SOF, Study of Osteoporotic Fractures. ^a^ *p* < 0.05 vs. robust; ^b^ *p* < 0.05 vs. pre-frail.

**Table 2:** Frequency (%) of distribution of older adults according to diet characteristics and frailty status.

FRAIL				mFP			SOF		
Variables	Robust (*n* = 22)	Pre-Frail (*n* = 126)	Frail (*n* = 52)	Robust (*n* = 15)	Pre-Frail (*n* = 154)	Frail (*n* = 31)	Robust (*n* = 27)	Pre-Frail (*n* = 127)	Frail (*n* = 46)
*Protein, g/kg*									
<0.8	0 (0.0)	8 (4.0)	3 (1.5)	0 (0.0)	10 (5.0)	1 (5.0)	0 (0.0)	7 (3.5)	4 (2.0)
≥0.8	22 (11.0)	118 (59.0)	49 (24.5)	14 (7.0)	144 (72.4)	30 (15.1)	27 (13.5)	120 (60.0)	42 (21.0)
*Protein, g/kg*									
<1.0	2 (1.0)	27 (13.5)	10 (5.0)	0 (0.0)	30 (15.1)	9 (4.5)	4 (2.0)	26 (13.0)	9 (4.5)
≥1.0	20 (10.0)	99 (49.5)	42 (21.0)	14 (7.0)	124 (62.3)	22 (11.1)	23 (11.5)	101 (50.5)	37 (18.5)
*Protein, g/kg*									
<1.2	10 (5.0)	39 (19.5)	16 (8.0)	2 (1.0)	50 (25.1)	12 (6.0)	12 (6.0)	38 (19.0)	15 (7.5)
≥1.2	12 (6.0)	87 (43.5)	36 (18.0)	12 (6.0)	104 (52.3)	19 (9.5)	15 (7.5)	89 (44.5)	31 (15.5)
*Protein, g/kg*									
<1.5	12 (6.0)	66 (33.0)	25 (12.5)	4 (2.0)	78 (39.2)	20 (10.1)	17 (8.5)	65 (32.5)	21 (10.5)
≥1.5	10 (5.0)	60 (30.0)	27 (13.5)	10 (5.0)	76 (38.2)	11 (5.5)	10 (5.0)	62 (31.0)	25 (12.5)
*Isoleucine, g*									
<4.4	12 (6.0)	62 (31.0)	25 (12.5)	5 (2.5)	67 (33.7)	26 (13.1)	17 (8.5)	56 (28.0)	26 (13.0)
≥4.4	10 (5.0)	64 (32.0)	27 (13.5)	9 (4.5)	87 (43.7)*	5 (2.5)*	10 (5.0)	71 (35.5)	20 (10.0)
*Leucine, g*									
<7.1	11 (5.5)	62 (31.0)	26 (13.0)	5 (2.5)	67 (33.7)	26 (13.1)	16 (8.0)	56 (28.0)	27 (13.5)
≥7.1	11 (5.5)	64 (32.0)	26 (13.0)	9 (4.5)	87 (43.7)*	5 (2.5)*	11 (5.5)	71 (35.5)	19 (9.5)
*Valine, g*									
<4.7	12 (6.0)	62 (31.0)	26 (13.0)	5 (2.5)	68 (34.2)	26 (13.1)	17 (8.5)	57 (28.5)	26 (13.0)
≥4.7	10 (5.0)	64 (32.0)	26 (13.0)	9 (4.5)	86 (43.2) *	5 (2.5) *	10 (5.0)	70 (35.0)	20 (10.0)
*CV*									
<0.38	6 (3.0)	52 (26.0)	18 (9.0)	6 (3.0)	57 (28.6)	13 (6.5)	6 (3.0)	53 (26.5)	17 (8.5)
0.38–0.45	16 (8.0)	71 (35.5)	32 (16.0)	8 (4.0)	94 (47.2)	16 (8.0)	21 (10.5)	71 (35.5)	27 (13.5)
>0.45	0 (0.0)	3 (1.5)	2 (1.0)	0 (0.0)	3 (1.5)	2 (1.0)	0 (0.0)	3 (1.5)	2 (1.0)
*≥0.4 g protein/kg/meal*									
0	1 (5.0)	15 (7.5)	8 (4.0)	1 (5.0)	19 (9.5)	4 (2.0)	1 (5.0)	16 (8.0)	7 (3.5)
1	14 (7.0)	74 (37.0)	28 (14.0)	9 (4.5)	84 (42.2)	22 (11.1)	19 (9.5)	72 (36.0	25 (12.5)
≥2	7 (3.5)	37 (18.5)	16 (8.0)	4 (2.0)	51 (25.6)	5 (2.5)	7 (3.5)	39 (19.5)	14 (7.0)
*≥30 g protein/meal*									
0	1 (5.0)	15 (7.5)	8 (4.0)	1 (5.0)	19 (9.5)	4 (2.0)	1 (5.0)	16 (8.0)	7 (3.5)
1	14 (7.0)	74 (37.0)	28 (14.0)	9 (4.5)	84 (42.2)	22 (11.1)	19 (9.5)	72 (36.0	25 (12.5)
≥2	7 (3.5)	37 (18.5)	16 (8.0)	4 (2.0)	51 (25.6)	5 (2.5)	7 (3.5)	39 (19.5)	14 (7.0)

Abbreviations: CV, coefficient of variation; mFP, modified frailty phenotype; SOF, Study of Osteoporotic Fractures; * Z-score ≥ 1.96 vs. participants with low intake in the same frailty category.
